# Range-Wide Sex-Chromosome Sequence Similarity Supports Occasional XY Recombination in European Tree Frogs (*Hyla arborea*)

**DOI:** 10.1371/journal.pone.0097959

**Published:** 2014-06-03

**Authors:** Christophe Dufresnes, Matthias Stöck, Alan Brelsford, Nicolas Perrin

**Affiliations:** 1 Department of Ecology and evolution, Biophore Building, University of Lausanne, Lausanne, Switzerland; 2 Leibniz-Institute of Freshwater Ecology and Inland Fisheries (IGB), Berlin, Germany; University of Uppsala, Sweden

## Abstract

In contrast with mammals and birds, most poikilothermic vertebrates feature structurally undifferentiated sex chromosomes, which may result either from frequent turnovers, or from occasional events of XY recombination. The latter mechanism was recently suggested to be responsible for sex-chromosome homomorphy in European tree frogs (*Hyla arborea*). However, no single case of male recombination has been identified in large-scale laboratory crosses, and populations from NW Europe consistently display sex-specific allelic frequencies with male-diagnostic alleles, suggesting the absence of recombination in their recent history. To address this apparent paradox, we extended the phylogeographic scope of investigations, by analyzing the sequences of three sex-linked markers throughout the whole species distribution. Refugial populations (southern Balkans and Adriatic coast) show a mix of X and Y alleles in haplotypic networks, and no more within-individual pairwise nucleotide differences in males than in females, testifying to recurrent XY recombination. In contrast, populations of NW Europe, which originated from a recent postglacial expansion, show a clear pattern of XY differentiation; the X and Y gametologs of the sex-linked gene *Med15* present different alleles, likely fixed by drift on the front wave of expansions, and kept differentiated since. Our results support the view that sex-chromosome homomorphy in *H. arborea* is maintained by occasional or historical events of recombination; whether the frequency of these events indeed differs between populations remains to be clarified.

## Introduction

Sex chromosomes have evolved along dramatically divergent pathways among vertebrates, depending on lineages. Most mammals and birds present strongly differentiated sex chromosomes, with highly degenerated Y and W chromosomes in males and females, respectively; in sharp contrast, many fishes, reptiles and amphibians present morphologically undistinguishable sex chromosomes. Two non-exclusive causes have been invoked to account for this homomorphy. On one hand, occasional turnovers may replace established sex chromosomes before they had time to decay (e.g. [1], [2]). On the other hand, occasional XY recombination may rejuvenate senescing Y chromosomes by purging the load of deleterious mutations that accumulate in non-recombining genomic regions [3]; very rare events of X-Y recombination seem sufficient to prevent Y degeneration [4].

The XY-recombination model recently received support from studies of European tree frogs. Several species of the *Hyla arborea* radiation inherited the same pair of sex chromosomes from a common ancestor (>5 Mya); despite arrest of recombination in males, the X and Y allelic sequences of sex-linked genes cluster by species, not by gametologs [5], [6], pointing to occasional events of recombination. Guerrero et al. [7] reached the same conclusion by analyzing with Approximate Bayesian Computations the patterns of XY divergence at sex-linked microsatellite loci. Surprisingly, however, no single event of male recombination could be detected by sibship analyses, despite thousands of offspring obtained in controlled crosses (e.g. [5], [6], [8]). Furthermore, West-European populations of the nominal species (*Hyla arborea*) consistently display sex-specific allelic frequencies at series of sex-linked microsatellite markers, often with male-diagnostic alleles (e.g. [5], [8]), pointing to the absence of XY recombination in their recent history.

**Figure 1:**
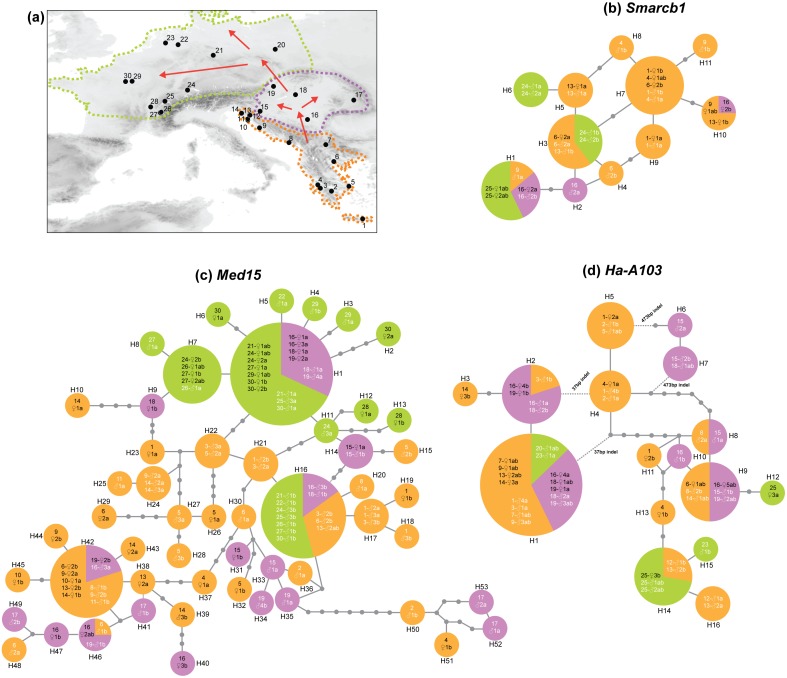
Sampling localities (a) and haplotype networks of *Smarcb1* (b), *Med15* (c) and *Ha-A103* (d). For each allele, labels indicate locality number, followed by the sex of the individual (black for females and white for males), a sample number, and the letter a or b (discriminating two alleles of heterozygotes; written “ab” for homozygotes). The colors of haplotypes correspond to the main phylogeographic regions across *H. arborea*’*s* present distribution range (as described by Stöck et al. 2012, and Dufresnes et al. 2013), delimited by thin dashed lines on the map (orange: southeastern European refugia; violet: Pannonian basin; green: NW Europe). Arrows show post-glacial recolonization routes.

To address this apparent paradox, we decided to extend investigations on the patterns of XY differentiation to a broader phylogeographic framework. West-European populations of *H. arborea* are of recent origin [9], [10]. The patterns of mitochondrial and nuclear diversity testify to a post-glacial expansion from southeastern Europe, where three main haplogroups (with ∼200 ky divergence) survived in distinct refugia across the Balkan Peninsula. While two of these mitochondrial lineages remained limited to the Balkans (Adriatic coast and southern Balkans; orange area in Fig. 1a), a third one expanded after the Last Glacial Maximum (∼15 kya) to recolonize first the Pannonian Basin (violet area), then from there Western and Northern Europe (green area), losing much of its diversity during this process [10]. In the present study, we use intraspecific sequence polymorphism of three sex-linked markers to seek evidence for possible events of XY recombination during this species’ late Pleistocene history.

## Methods

### DNA Sampling and Study Animals

DNA was sampled in 91 adult *H. arborea* (37 females, 54 males) using non-invasive buccal swabs in 87 live individuals or ethanol-preserved tissues in four collection specimens, and extracted manually (DNeasy kit, Qiagen) or with the Qiagen Biosprint robotic workstation. Samples were chosen to represent populations from the entire distribution range, from southern Greece to Western Europe (Fig. 1a, Table S1, Supporting Information). Live individuals were captured during the breeding period, and could be sexed unambiguously through secondary sexual traits; males being characterized by dark vocal sacs, nuptial excrescences, and mating calls. The four collection specimens were also sexed unambiguously by anatomical observation of either ovaries or testes. Our study was approved by the relevant Institutional Animal Care and Use Committee (IACUC), namely the Service de la Consommation et des Affaires Vétérinaires du Canton de Vaud (Epalinges, Switzerland); no live animal was sacrificed for the study (sampling only using non-invasive buccal swabs); other samples came from scientific collections specified in Table S1, Supporting Information.

### PCR Amplification, Cloning and Sequencing

We amplified three sex-linked markers: parts of the transcription co-factor *Med15* (including two exons and two introns; n = 55), the non-coding *Ha-A103* (n = 33), and one intron from the gene *Smarcb1* (n = 16), as described [5], [11]. Except for four *Smarcb1* samples, which we sequenced directly, all PCR products were cloned using the Promega pGEM-T Easy or Invitrogen TOPO-TA cloning kits. In most cases, at least eight clones per sample could be sequenced on an ABI3730 (Applied Biosystems), and consensuses were produced with Seaview [12]. When fewer than eight clones were obtained (24 cases) individuals were considered homozygous if at least six clones were identical. Two individuals only yielded three identical clones; we labeled the other allele as missing data and discarded these two individuals from the pairwise distance analyses. For directly sequenced *Smarcb1*, haplotypes could be reconstructed manually: two individuals were homozygous, and the other two shared identical genotypes with closely related alleles (differing by two polymorphic sites).

### Data Analyses

Sequences were manually aligned in Seaview, and analyzed by statistical parsimony networks with TCS (v. 1.21 [13]; parsimony limit set to 95%), which allows taking indel polymorphisms into account (option “gap as fifth base”). In addition to SNPs, *Med15* and *Ha-A103* contain microsatellite-like repeats [5], which were re-coded so that one tandem repeat difference corresponds to one mutational step. To confirm proper amplification and observed length differences within *Med15* and *Ha-A103*, we compared this data with results from microsatellite genotyping at these loci (details in [5], [8]). We followed the same re-coding strategy for two large indels (37 bp and 473 bp) within *Ha-A103* (Fig. 1d).

Although the two copies of a male necessarily comprise an X and a Y allele, it was *a priori* not possible to assign any specific copy to either the X or the Y chromosome (except for *Med15* in NW Europe populations; see Results and Discussion). Thus, X-Y and X-X divergence were compared by measuring, for each marker, the pairwise nucleotide differences *p_d_* (in DnaSP v. 5 [14]) between the two copies of every male (X-Y difference) and female (X-X difference). Comparisons between sexes and regions were assessed by analyses of variance (ANOVA) of *p_d_*, using a non-parametric permutation procedure to test the significance of differences (10′000 replicates; performed in *R* 2.13.1 [15]). The geographic regions were defined according to the phylogeographic structure of the species as inferred by multilocus nuclear and mtDNA data by Dufresnes et al. [10].

## Results and Discussion

Sequences (Alignment S1, Supporting Information) of *Med15* (992 bp) were the most polymorphic (64 variable sites, 33 parsimony-informative), followed by *Ha-A103* (504 bp without a 473 bp indel found in 2 individuals, 19 variable sites, 15 parsimony-informative) and *Smarcb1* (411 bp, 8 variable sites, all parsimony-informative). Most of the diversity was found in southeastern Europe, and the Pannonian Basin (Fig. 1: orange and violet areas); for all three markers, postglacial populations from Northern and Western Europe (green area) harbor fewer haplotypes. These results corroborate evidence from autosomal and mitochondrial data of a large-scale post-glacial colonization of the Pannonian Basin from several glacial refugia in southeastern Europe, namely the southern Balkans and the Adriatic coast, followed by a later expansion to Western and Northern Europe, during which much variance was [10].

Haplotypic networks (Fig. 1b–d) show a generally large mix of male and female alleles. Averaged over all individuals and populations, alleles were not more diverged in males (X-Y) than in females (X-X) at all three loci (for *Ha-A103*: F_1,31_ = 0.43, p-value = 0.55; for *Smarcb1*: F_1,14_ = 0.14, p-value = 0.72; for *Med15* (F_1,51_ = 0.44, p-value = 0.50). Many males presented identical X and Y haplotypes, or shared both of their alleles with females (Fig. 1b–d). Interestingly, two large indels in *Ha-A103* were shared by X and Y haplotypes: the same 37 bp deletion occurred on the two copies of males from southeastern Europe (e.g. 3-♂1, 7-♂1 and 9-♂3), the Pannonian Basin (e.g. 18-♂2, 19-♂3) and NW Europe (20-♂1); similarly, the same 473 bp insertion was shared by the X and Y alleles of two males from the Pannonian Basin (15-♂2 and 18-♂1). Given that *Ha-A103* (as well as *Med15*) display perfect lineage sorting by species in *Hyla arborea, H. molleri, H. intermedia* [5], these patterns cannot be accounted for by the maintenance of ancestral polymorphisms predating speciation, and thus provide clear evidence for XY recombination events, postdating species divergence.

**Figure 2:**
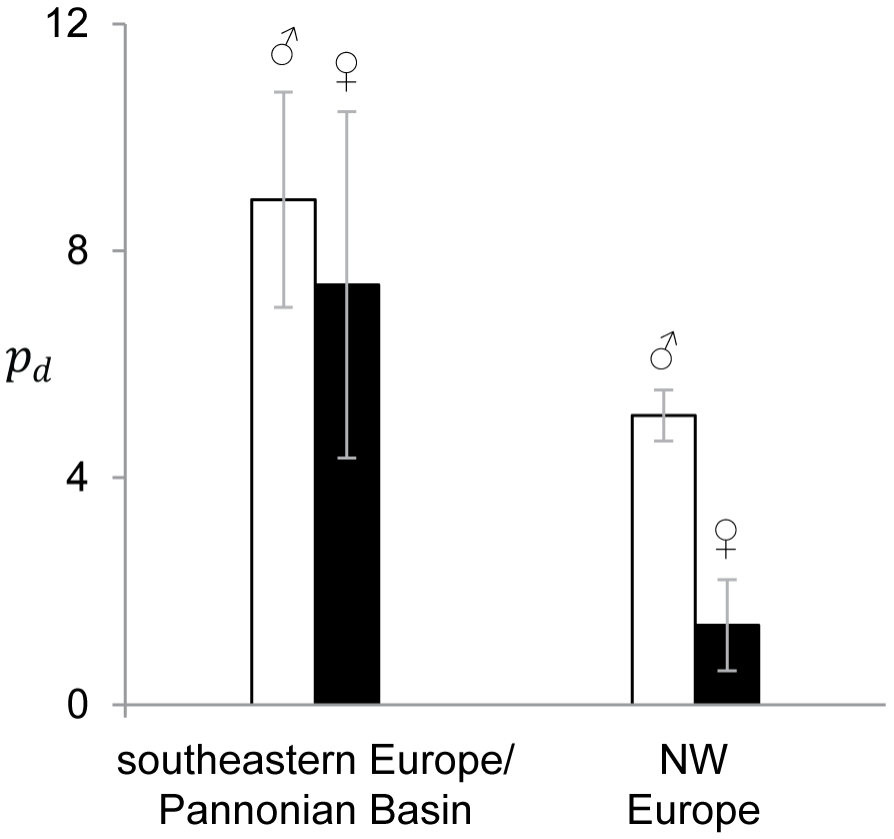
Pairwise nucleotide differences (*p_d_*) between the two *Med15* alleles of every male (white bars) and female (black bars). Larger values are found in the southeastern Europe and the Pannonian Basin (left) than in NW Europe (right), and, in the latter region, in males than in females.

However, closer inspection reveals some differences between phylogeographic regions. In particular, the patterns of diversity and differentiation at *Med15* display a clear trend with geography (Fig. 1c). Whereas male alleles from southeastern refugial (orange) and Pannonian (violet) populations mix randomly with female alleles in the network, sex-differences occur for NW Europe (green, loc. 20–30). With the single exception of individual 29-♂1, all males harbor one and only one copy of allele H_16_, otherwise identified from sibship analyses as the Y allele in several Swiss and French populations (referred to as allele *Ha5-22* 236 in [8], [16]. This allele was only found in males throughout the *H. arborea* range covered by our study, including some from the Pannonian Basin (violet) and southeastern European glacial refugia (orange), where it might also occur on the Y. The second allele (i.e., the X copy) from all Western and Northern European *Hyla* males belongs to the H_1_–H_15_ haplogroup, otherwise shared by all females from these populations, plus some from the Pannonian Basin. This was the only haplogroup found in females from Western and Northern Europe, but many more segregated in females from the Pannonian Basin and southeastern European refugial populations. Accordingly, a two-way ANOVA, with sex and geographic region (North-Western Europe *versus* Pannonian and southeastern populations) as factors, identified significant effects for both the region (F_1,49_ = 12.5, p-value = 0.009) and the interaction between sex and region (F_1,49_ = 3.38, p-value = 0.023). As shown in Fig. 2, this reflects respectively the higher diversity of southeastern European and Pannonian populations (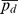 = 8.2 as opposed to 3.3 in Northern and Western Europe) and the strong XY differentiation in North-Western Europe (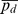 = 5.1 in males *versus* 1.4 in females) compared to the rest of the range (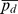 = 8.9 in males *versus* 7.4 in females). The only exception (male 29-♂1, from the westernmost part of the distribution range) deserves special mention: given the strong differentiation between the haplotypes H_3_ and H_4_ harbored by this male on one hand, and the haplotype H_16_ fixed on the Y of other males on the other hand, the pattern observed is most likely to result from a recent (post-glacial) event of XY recombination.

No such interaction was identified for *Ha-A103* and *Smarcb1*, which might however result from a lower power due to smaller sample sizes, and to lower levels of polymorphism. Markers *Ha-A103* and *Smarcb1* were clearly less variable than *Med15,* and our range-wide samples appear to represent most of their diversity. The XY overlap found in NW Europe populations for these two markers are compatible with post-glacial XY recombination, but might also stem from ancestral polymorphisms shared by the X and Y chromosomes that contributed to the post-glacial expansion towards NW Europe.

## Conclusion

Our study provides range-wide empirical evidence that X and Y chromosomes have exchanged genetic material until recently, and are possibly still recombining occasionally in the southern part of *H. arborea* geographic range. Although our results solve the apparent paradox mentioned in the Introduction, they also raise new questions, by suggesting that XY recombination rate might vary phylogeographically, being higher in refugial populations than in post-glacial-origin populations of NW-Europe. Patterns at *Med15* in particular suggest that different X and Y alleles have been fixed by drift in the wave of expanding populations, and were maintained differentiated over the last 10 ky. Whether the amount of recombination significantly between regions, however, remains to be clarified. More accurate reconstruction of the phylogeography of Y haplotypes, using a larger number of fast-evolving markers, should help to better characterize the spatial and temporal dynamics of recombination episodes in *H. arborea*.

## Supporting Information

Table S1 Detailed sample information, including origin, nature, sex, number of clones sequenced and GenBank accession numbers (provided upon acceptance of the paper).
(XLSX)

Alignment S1 Alignments of all three sequences markers (*Ha-A103*, *Med15* and *Smarcb1*) are provided along with this paper as Supporting Information.
(ZIP)
